# Targeting MAPK/NF-κB Pathways in Anti-Inflammatory Potential of Rutaecarpine: Impact on Src/FAK-Mediated Macrophage Migration

**DOI:** 10.3390/ijms23010092

**Published:** 2021-12-22

**Authors:** Thanasekaran Jayakumar, Kao-Chang Lin, Chao-Chien Chang, Chih-Wei Hsia, Manjunath Manubolu, Wei-Chieh Huang, Joen-Rong Sheu, Chih-Hsuan Hsia

**Affiliations:** 1 Graduate Institute of Medical Sciences, College of Medicine, Taipei Medical University, Taipei 110, Taiwan; jayakumar@tmu.edu.tw (T.J.); gaujang@mail2000.com.tw (K.-C.L.); d119106003@tmu.edu.tw (C.-W.H.); d119110003@tmu.edu.tw (W.-C.H.); 2 Chi Mei Medical Center, Department of Neurology, Tainan 710, Taiwan; 3 Department of Pharmacology, School of Medicine, College of Medicine, Taipei Medical University, Taipei 110, Taiwan; cgh05761@cgh.org.tw; 4 Department of Cardiovascular Center, Cathay General Hospital, Taipei 106, Taiwan; 5 School of Medicine, College of Medicine, Fu Jen Catholic University, New Taipei City 242, Taiwan; 6 Department of Evolution, Ecology and Organismal Biology, Ohio State University, Columbus, OH 43212, USA; manubolu.1@osu.edu; 7 Translational Medicine Center, Shin Kong Wu Ho-Su Memorial Hospital, Taipei 111, Taiwan

**Keywords:** rutaecarpine, anti-inflammation, PI3K/Akt, MAPK, NF-κB, Src/FAK, cell migration, molecular mechanism

## Abstract

Studies have discovered that different extracts of *Evodia rutaecarpa* and its phytochemicals show a variety of biological activities associated with inflammation. Although rutaecarpine, an alkaloid isolated from the unripe fruit of *E. rutaecarpa*, has been exposed to have anti-inflammatory properties, the mechanism of action has not been well studied. Thus, this study investigated the molecular mechanisms of rutaecarpine (RUT) in lipopolysaccharide (LPS)-induced RAW 264.7 macrophages. RUT reserved the production of nitric oxide (NO) and the expression of inducible nitric oxide synthase (iNOS), cyclooxygenase-2 (COX-2), tumor necrosis factor (TNF-α), and interleukin (IL)-1β in the LPS-induced macrophages. RUT showed an inhibitory effect on the mitogen-activated protein kinases (MAPKs), and it also inhibited nuclear transcription factor kappa-B (NF-κB) by hindering IκBα and NF-κB p65 phosphorylation and p65 nuclear translocation. The phospho-PI3K and Akt was concentration-dependently suppressed by RUT. However, RUT not only suggestively reduced the migratory ability of macrophages and their numbers induced by LPS but also inhibited the phospho-Src, and FAK. Taken together, these results indicate that RUT participates a vital role in the inhibition of LPS-induced inflammatory processes in RAW 264.7 macrophages and that the mechanisms involve PI3K/Akt and MAPK-mediated downregulation of NF-κB signaling pathways. Notably, reducing the migration and number of cells induced by LPS via inhibiting of Src/FAK pathway was also included to the anti-inflammatory mechanism of RUT. Therefore, RUT may have potential benefits as a therapeutic agent against chronic inflammatory diseases.

## 1. Introduction

Inflammation is a physiological event of an organism which protects from chemical, physical, infectious agents, environmental toxins, ischemia, or an antigen-antibody interaction. Nevertheless, chronic inflammation may induce tissue damage. In the United States, every year, more than 500,000 patients suffer from sepsis activated by severe systemic inflammation [1]. A variety of external or internal stimulators, such as calcium homeostasis, platelet-activating factor, cytokines, interleukins (IL), chemotaxis, cyclooxygenase (COX), adhesion molecules, reactive oxygen species (ROS), and nitric oxide (NO), are involved in inflammation. NSAIDs (Non-steroidal anti-inflammatory drugs) are the generally used therapeutic drugs for managing of inflammatory diseases as they play a significant role among anti-inflammatory drugs [2,3]. Ibuprofen and aspirin have developed among NSAIDs as potential anti-inflammatory drugs that could effectively suppress the role of COX enzyme and inhibit prostaglandin synthesis [4,5]. On the other hand, prolonged intake of NSAIDs is associated with a variety of side effects, including bleeding, coagulopathy, interstitial nephritis, gastrointestinal mucosa damage, and allergic reactions, because of their very low prostaglandin synthesis [6,7].

**Figure 1:**
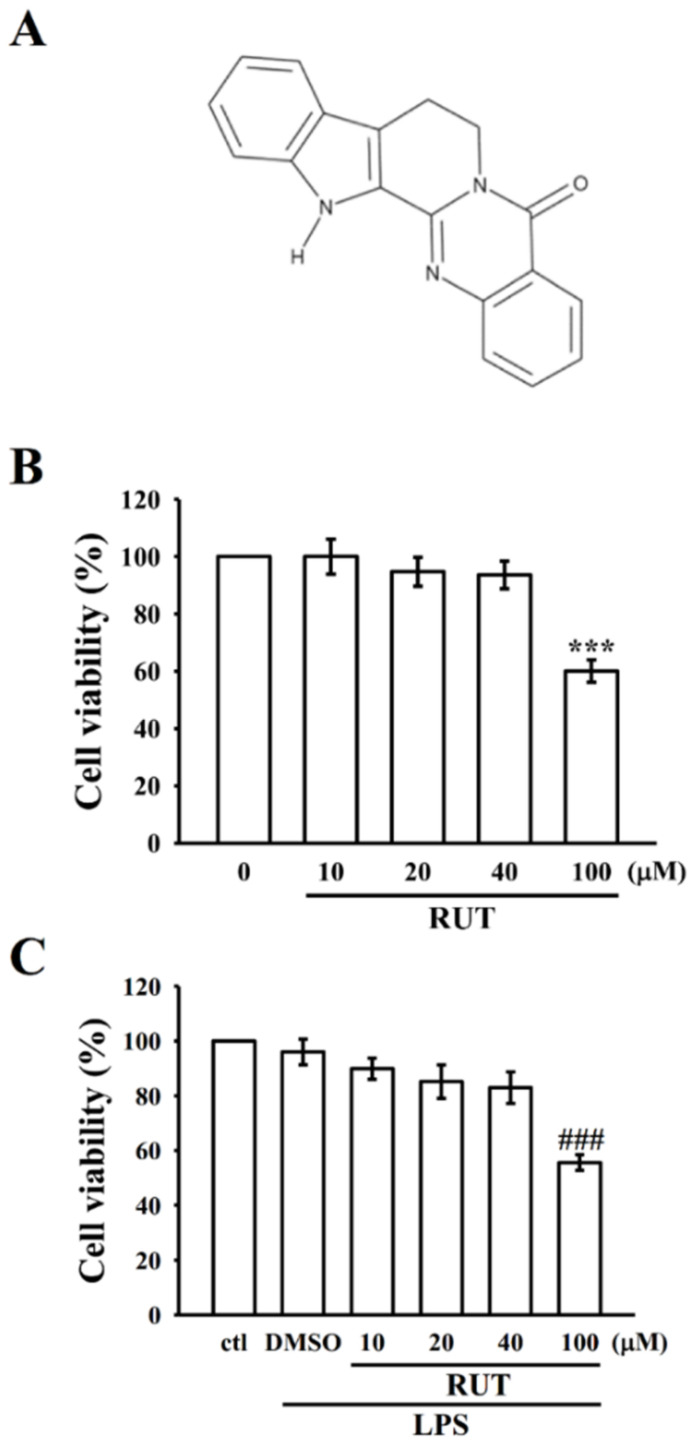
Effects of rutaecarpine (RUT) on cell viability in lipopolysaccharide (LPS)-stimulated RAW 264.7 cells. (**A**) Chemical structure of RUT. (**B**) Cells were treated with 0.1% DMSO or pretreated with RUT (10–100 μM) for 24 h. (**C**) Cells were treated with 0.1% DMSO or pretreated with RUT (10–100 μM) for 20 min and then treated with LPS (1 μg/mL) for 24 h. Cell viability was evaluated as described in the Methods section. Data are presented as the means ± SEM (*n* = 4). *** *p* < 0.001, compared with the control group; ^###^ *p* < 0.001, compared with the LPS group.

Recently, natural compounds have been regarded as the amplest sources for novel drug development. For instance, aspirin and corticosterone are the two commonly used natural anti-inflammatory drugs. Flavonoids have long been used as promising therapeutic agents for the treatment of inflammatory diseases [8]. Herbal medicines are prevalently used as alternative medicines. For example, the fruit of *Evodia rutaecarpa* has long been used in herbal remedies for the treatment of gastrointestinal disorders, headache, amenorrhea, and postpartum hemorrhage in traditional oriental medicine [9,10,11]. Rutaecarpine (8,13-dihydro-7H-indolo-[2′,3′:3,4]-pyrido [2,1-b]-quinazolin-5-one; Figure 1A) is an alkaloid firstly isolated from *E. rutaecarpa* [12].

Studies have established that rutaecarpine (RUT) owns widespread biological and pharmacological properties, such as diuresis, perspiration, uterotonic action, improvement of cerebral functions, antinociception, and anti-obesity [13,14]. This compound shows potential effect on inhibiting platelet aggregation induced by various agonists [14,15,16]. Our recent study has also demonstrated the antiplatelet mechanisms of RUT via downregulating the PI3K/Akt and MAPK signaling cascades [17]. Rutaecarpa and its bioactive components display anti-inflammatory activities by their different abilities for constraining iNOS-dependent NO production in activated inflammatory neutrophils and microglial cells. Though RUT has suggested to have antinociceptive activity via mediated by its anti-inflammatory action [18], the detailed molecular mechanism has not been explored sufficiently. In this milieu, we have methodically investigated the molecular mechanism of RUT in lipopolysaccharide (LPS)-stimulated macrophages.

## 2. Results

### 2.1. RUT Reversed LPS-Induced Inflammatory Mediators and Cytokines without Inducing Cellular Toxicity

**Figure 2:**
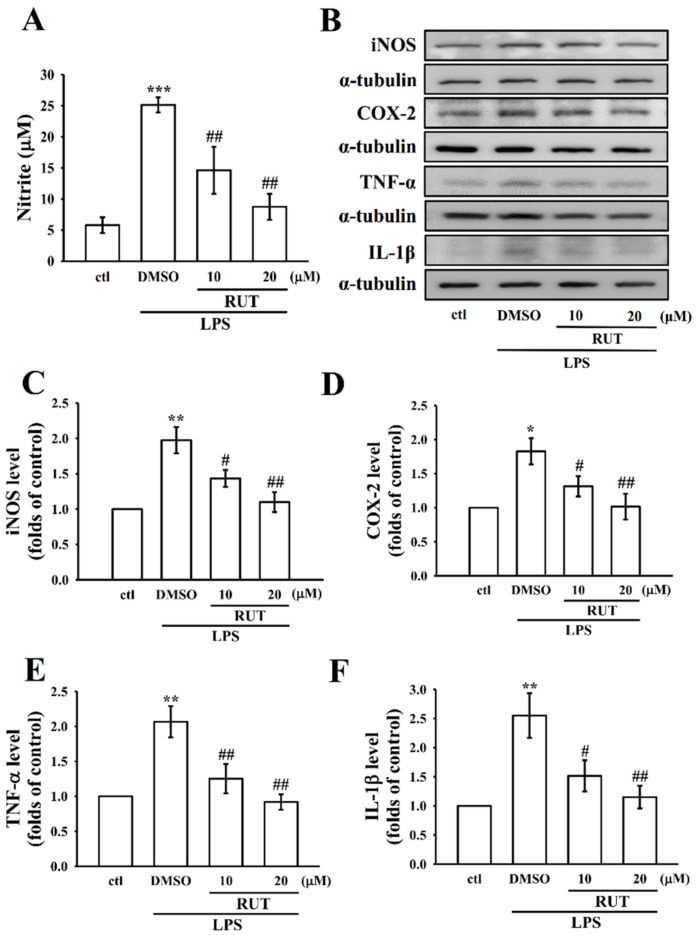
Effects of RUT on nitric oxide (NO) production, and the expression of inducible nitric oxide synthase (iNOS), cyclooxygenase-2 (COX-2), tumor necrosis factor alpha (TNF-α), and interleukin-1 beta (IL-1β) in LPS-stimulated RAW cells. Cells were pretreated with RUT (10 and 20 μM) for 20 min and then stimulated by LPS (1 μg/mL) for 24 h. (**A**) NO was measured using Griess reagent. (**B**–**F**) The levels of (**C**) iNOS, (**D**) COX-2, (**E**) TNF-α, and (**F**) IL-1β protein expression were evaluated as described in the Methods section. Data are presented as the means ± SEM (*n* = 4); * *p* < 0.05, ** *p* < 0.01, and *** *p* < 0.001, compared with the control group; ^#^ *p* <0.05 and ^##^ *p* < 0.01, compared with the LPS group.

As shown in Figure 1B,C, RUT alone or with LPS did not induce cytotoxic effect up to the concentrations of 10–40 μM for 24 h in RAW 264.7 cells. To examine the potency of RUT on the LPS-induced inflammatory mediators in RAW cells, the production of NO and the expression of iNOS, COX-2, TNF-α, and IL-1β were investigated. In comparison to the control cells, LPS could expressively induce inflammatory mediators of NO (*p* < 0.001), iNOS (*p* < 0.01), and COX-2 (*p* < 0.05) and cytokines of TNF-α and IL-1β (*p* < 0.01)). Compared with the LPS group, 10 and 20 μM of RUT momentously suppressed the level of NO (*p* < 0.01) and the expression of iNOS, COX-2 (*p* < 0.05 and 0.01), TNF-α (*p* < 0.01), and IL-1β (*p* < 0.05 and *p* < 0.01) in a concentration-dependent manner (Figure 2A–F). This result indicates that RUT owns its anti-inflammatory effects via modulating inflammatory mediators without causing cytotoxicity in RAW cells.

### 2.2. RUT Regulated PI3K/Akt, MAPK, and NF-κB Signaling Pathways in RAW264.7 Cells

Based on the reputation and the pivot genes of the network, PI3K/Akt, MAPK signaling pathways, as well as nuclear factor NF-κB p65, were selected for experimental justification as major inflammation related signaling pathways in RAW cells. It has also been proposed that inhibition of the MAPK and NF-kB pathways could considerably reduce the release of several inflammatory cytokines in macrophages stimulated by LPS. Equally, since RUT has potential effect on inhibiting LPS-induced cytokines and inflammatory mediators, as shown in Figure 2, we investigated the probable mechanism of RUT by investigating the following signaling molecules:

#### 2.2.1. RUT Greatly Recovered LPS Induced MAPKs Signaling Proteins

**Figure 3:**
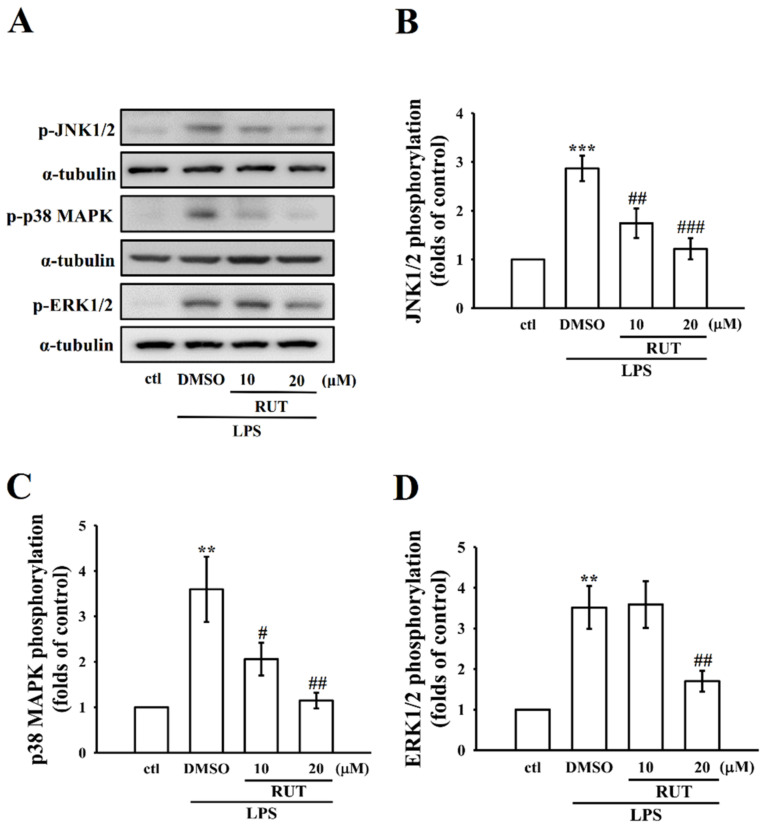
Influence of RUT on LPS-induced phosphorylation of c-Jun NH2-terminal kinase (JNK), p38 mitogen-activated protein kinase (p38 MAPK), and extracellular signal-regulated kinase (ERK) in RAW cells (**A**–**D**). Cells were treated with 0.1% DMSO or RUT (10 and 20 μM) for 20 min, followed by LPS (1 μg/mL) for 30 min, and the phosphorylation of (**B**) JNK, (**C**) p38 MAPK, and (**D**) ERK were evaluated by immunoblotting assay as described in the Methods. Data are presented as the means ± SEM (*n* = 4). ** *p* < 0.01 and *** *p* < 0.001, compared with the control group; ^#^ *p* < 0.05, ^##^ *p* < 0.01, and ^###^ *p* < 0.001 compared with the LPS group.

To recognize the regulation of survival and differentiation for several types of cells, MAPKs play a significant factor, as they are nearly associated with different cellular responses [19]. As shown in Figure 3A–D, at the ideal concentrations of 10 and 20 μM, RUT expressively decreased LPS induced phosphorylated protein expression of p38 MAPK (*p* < 0.05; *p* < 0.01) and JNK (*p* < 0.01; *p* < 0.001) in a concentration-dependent manner in comparison with the LPS untreated group; however, it has inhibited ERK (*p* < 0.01) phosphorylation only at its higher concentration of 20 μM. Thus, in comparison with the effects on p38 MAPK and JNK, RUT shows less potential inhibitory effect on ERK phosphorylation.

#### 2.2.2. RUT Modulates Phosphoinositide 3-Kinase (PI3K) and Akt Pathway

**Figure 4:**
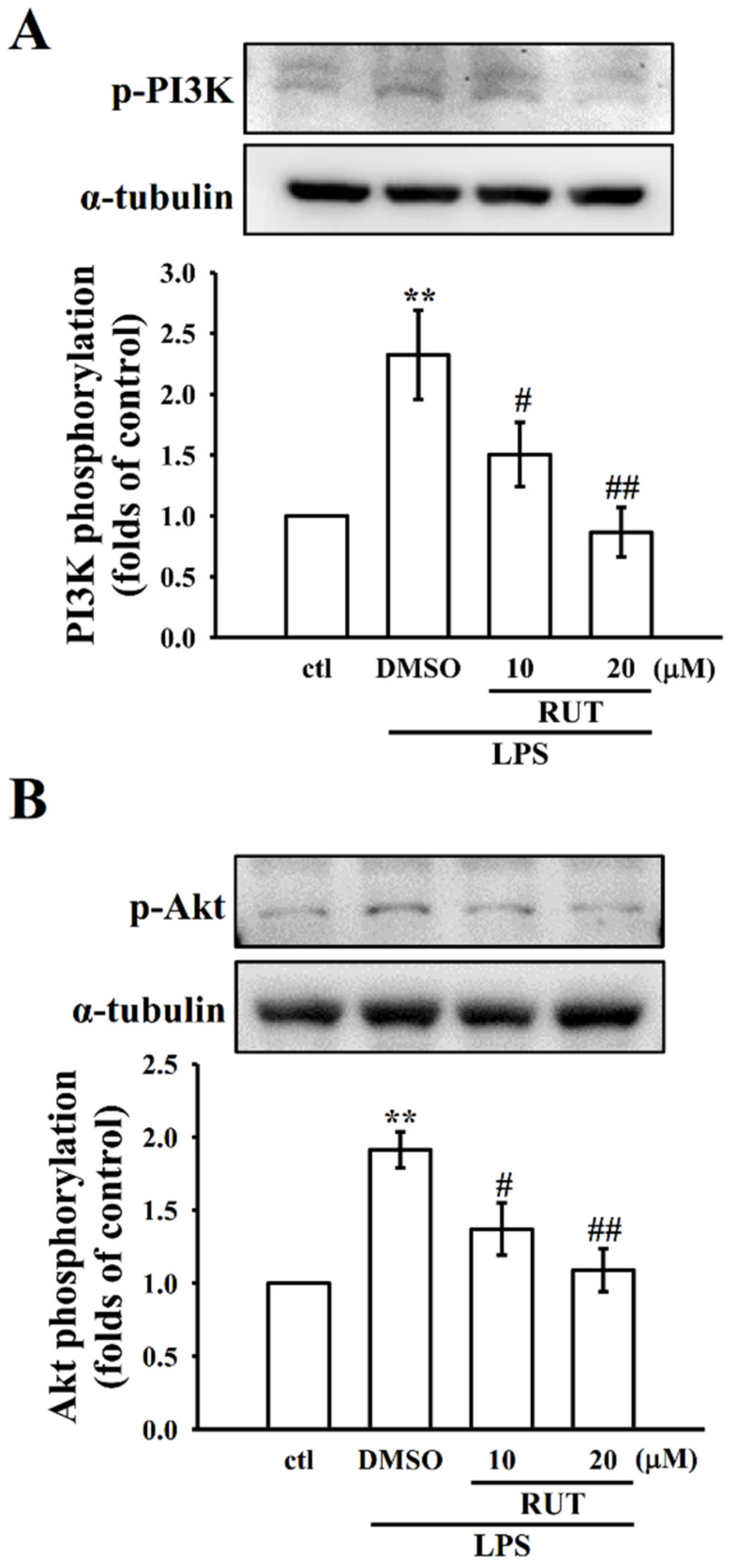
Effect of RUT on LPS-induced phosphoinositide 3-kinase (PI3K)/Akt signaling pathways in RAW cells. RAW cells were treated with 0.1% DMSO or RUT (10 and 20 μM) for 20 min, followed by LPS (1 μg/mL) for 30 min. The expression of phosphorylated (**A**) PI3K and (**B**) Akt were detected by immunoblotting. Data are expressed as the mean ± SEM (*n* = 4). ** *p* < 0.01, compared with the control group; ^#^ *p* < 0.05 and ^##^ *p* < 0.01, compared with the LPS group.

In different types of cells, PI3K-Akt signaling pathway facilitates proliferation, apoptosis, differentiation, and migration. It is well acknowledged that PI3K/Akt signaling pathway plays role on the regulation of pro-inflammatory genes inducing NF-κB activity [20]. A previous study observed that LPS-induced NF-κB activation directly controlled by the phosphorylation of PI3K/Akt in microglial cells [21]. Hence, the PI3K/Akt signaling pathway is a promising target for therapeutic intervention to control inflammatory diseases. For this reason, this study examined if RUT has an impact on the regulation of PI3K/Akt phosphorylation, and the results show that the phosphorylated levels of PI3K and Akt were significantly (*p* < 0.01) increased after LPS treatment. This increased phosphorylation of PI3K/Akt was expressively reduced in cells that had been pretreated with RUT at 10 (*p* < 0.05) and 20 (*p* < 0.01) μM before LPS stimulation in a concentration-dependent manner (Figure 4A,B), indicating that these molecules may play a role in the treatment of inflammatory diseases by directing appropriate signaling pathways.

#### 2.2.3. Effects of RUT on Nuclear Factor Kappa B (NF-κB) Signaling Pathways

**Figure 5:**
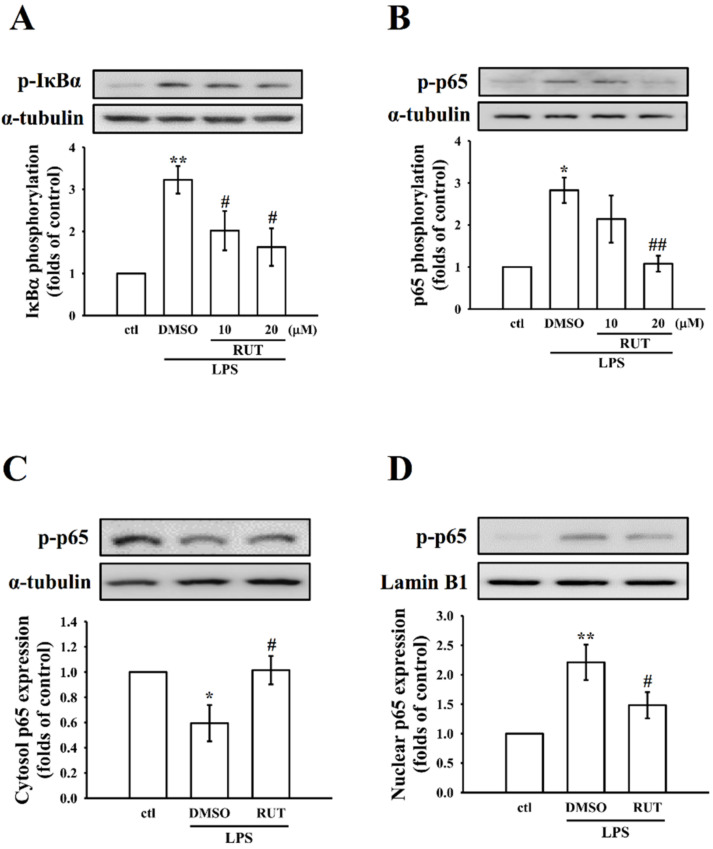
Effects of RUT on LPS-induced IκBα and p65 phosphorylation, and nuclear translocation of NF-κB p65 in RAW cells. (**A**,**B**) Cells were treated with 0.1% DMSO or RUT (10 and 20 μM) for 20 min, followed by LPS (1 μg/mL) for 30 min. The phosphorylation of (**A**) IκBα and (**B**) p65 were determined by immunoblotting. (**C**,**D**) Cells were treated with 0.1% DMSO or RUT (20 μM) for 20 min, followed by LPS (1 μg/mL) for 30 min. The cytosolic and nuclear fractions were isolated using the NE-PER kit and then subjected to Western blotting to detect p65 expression. α-tubulin and Lamin B1 were used as internal controls for the nucleus and cytosol, respectively. Data are presented as the means ± SEM (*n* = 4). * *p* < 0.05 and ** *p* < 0.01, compared with the control group; ^#^ *p* < 0.05 and ^##^ *p* < 0.01, compared with the LPS group.

**Figure 6:**
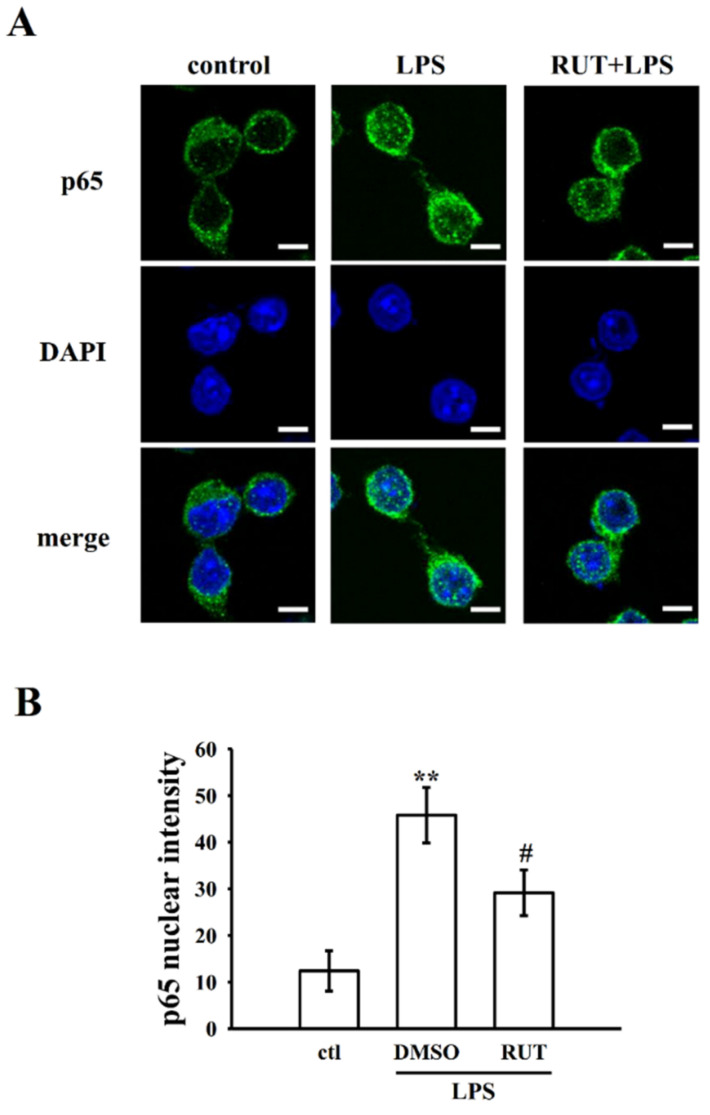
Effects of RUT on LPS induced NF-κB p65 nuclear translocation in RAW cells. Cells were treated with 0.1% DMSO, RUT (20 μM) for 20 min, followed by LPS (1 μg/mL) for 30 min. (**A**) The immunofluorescence staining analysis was performed with an anti-p65 antibody and FITC-conjugated anti-rabbit IgG antibody (green). 4′,6-diamidino-2-phenylindole (DAPI) was used to label the nuclei (blue). The images were captured by confocal microscopy (scale bar = 5 μm). (**B**) Data were graphed by pooling multiple images, with each individual data point corresponding to the mean fluorescence intensity of each individual cell nucleus. Data are presented as the means ± SEM (*n* = 4). ** *p* < 0.01, compared with the control group; ^#^ *p* < 0.05, compared with the LPS group.

In the activated macrophages, LPS could trigger NF-κB activation, which control several inflammatory cascades through NO, iNOS, TNF-α, IL-6, and other inflammatory mediators production [22]. Therefore, we analyzed the effect of RUT in LPS-induced NF-κB signaling, such as the phosphorylation of IκBα, and phosphorylation and nuclear translocation of p-p65. As labeled in Figure 5A,B, phosphorylation of IκBα (Figure 5A, *p* < 0.01), p65 (Figure 5B, *p* < 0.05)) and p65 cytosolic (*p* < 0.05), and nuclear (*p* < 0.01) translocation (Figure 5C,D) were augmented in LPS-induced cells when compared with normal cells. Attractively, pretreatment of cells with RUT at 10 and 20 μM inhibited the elevations of IκBα (*p* < 0.05) and p65 (*p* < 0.01) phosphorylation; moreover, it has effect on inhibiting the expression p65 in the nuclear fraction, whereas elevating in the cytosolic fraction of cells. Moreover, the inhibitory effect of RUT on p65 nuclear translocation was further evidenced by a confocal image analysis, as shown in Figure 6. The results indicate that LPS induced an increased FITC labeled NF-κB p65 (green fluorescence) in the nucleus of RAW cells. However, RUT pretreatment significantly reduced this green fluorescence staining in the nuclear fraction (Figure 6A,B). Overall, these results indicate that the anti-inflammatory activity of RUT appears to be mediated by the inhibition of the inflammatory mediators, including NO, iNOS, TNF-α, IL-1β, and COX2, as well as the regulation of MAPK, NF-κB p65, and PI3K/Akt signaling pathways.

### 2.3. RUT Suppresses RAW Cell Migration

**Figure 7:**
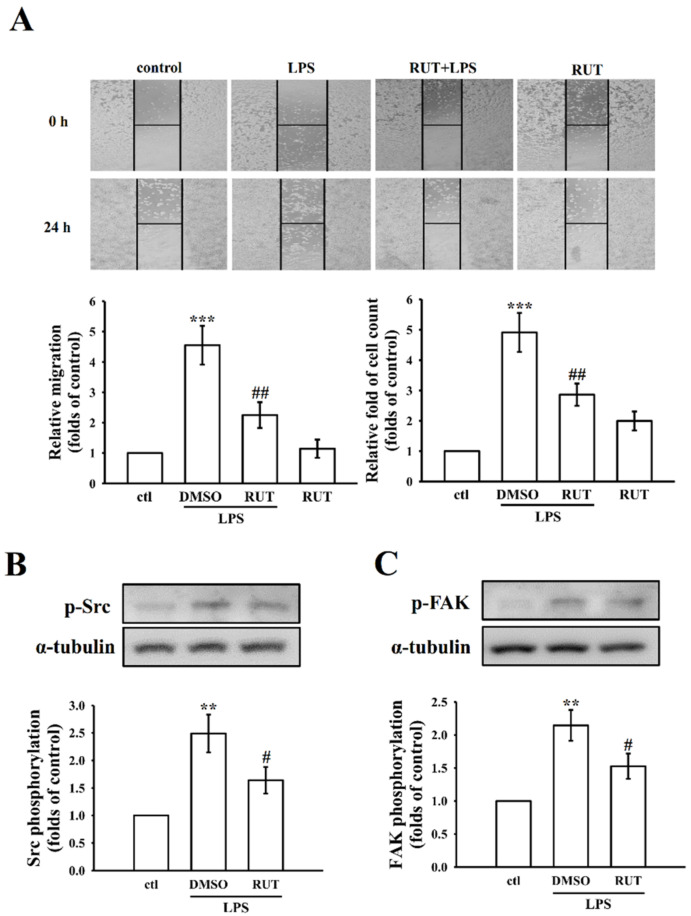
Effects of RUT on migration and Src/FAK expression in LPS-treated RAW cells. (**A**–**C**) Cells were treated with 0.1% DMSO or RUT (20 μM) for 20 min, followed by LPS (1 μg/mL) for 24 h. Cell migration was measured by scratch wound healing assay. (**A**) Images of cell migration at 0 and 24 h. (**B**) Src and (**C**) FAK phosphorylation were determined by immunoblotting. Data are presented as the means ± SEM (*n* = 4). ** *p* < 0.01 and *** *p* < 0.001, compared with the control group; ^#^ *p* < 0.05 and ^##^ *p* < 0.01, compared with the LPS group.

LPS is well recognized to provoke a variety of cellular activities, including cell migration in macrophages [23]. To study the effect of RUT on macrophage migration, the migratory potential of RAW 264.7 macrophages treated without or with RUT prior to LPS stimulation was determined. As shown in Figure 7A, compared to control cells, significantly (*p* < 0.001) augmented migration and increased cell count was found in LPS stimulated RAW macrophages. Interestingly, LPS-induced cell migration and their numbers was remarkably (*p* < 0.01) reversed in RUT (20 µM) treated cells for 24 h. Fascinatingly, RUT treatment alone showed almost a similar migration level to that of normal cells (Figure 7A). It indicates that RUT, without inducing cell toxicity, potently inhibits macrophage migration.

### 2.4. RUT Inhibits LPS-Induced Src/FAK Activation in RAW Cells

This study further examined if RUT suppresses macrophage migration by inhibiting the phosphorylation of Src/FAK proteins, since a study found that inhibition of macrophage migration by a flavonoid compound, quercetin is associated with FAK-paxillin pathway and cell motility [24]. As our assumption, in comparison with the untreated control RAW cells, LPS significantly increased the phosphorylation of FAK and Src (*p* < 0.01). This is consistent with the results found by Deramoudt [24]. As shown in Figure 7B,C, RUT pretreatment at 20 µM significantly (*p* < 0.05) reduced this increment, similar to the effect on the migration of RAW cells shown in Figure 7A.

## 3. Discussion

The results of this study demonstrated that the anti-inflammatory effect of rutaecarpine is responsible for the inhibition of inflammatory mediators, including NO production, iNOS, COX-2, IL-1β, and TNF-α expression, in LPS-induced RAW cells. Depending on the network pharmacology, MAPK, NF-κB, and PI3K/Akt was found to be the potential identified targets. The molecular mechanism of RUT on these targets was further verified by western blot and confocal image analysis. FAK is found to a distinguished substrate of Src; thus, Src-induced FAK phosphorylation acted as vital player in macrophage migration [25]. Therefore, to establish the participation of this signaling pathway in LPS-induced macrophage migration, the phosphorylated status of Src and FAK were examined in RAW cells. Fascinatingly, RUT administration in LPS-induced RAW cells significantly regulated all these MAPK, NF-κB, and PI3K-Akt signaling pathways. In addition, remarkably, RUT-mediated reduction of Src and FAK phosphorylation could be attributable to the blocked level of its corresponding macrophage migration.

Inducible nitric oxide synthase (iNOS) and cyclooxygenase-2 (COX-2) expression are markedly raised during the inflammatory events, which enhances the production of NO and PGE2, respectively. TNF-α provokes several inflammatory diseases, including cachexia, cytotoxicity, and sepsis, and IL-1β is believed to be associated in LPS-stimulated inflammatory processes. Activation of these inflammatory mediators causes cell and injury, resulting in numerous physiological ailments related with inflammation, such as chronic hepatitis and rheumatoid arthritis [26,27]. Therefore, these pro-inflammatory mediators might be the potential indicators for effective anti-inflammatory substances. This study shows that RUT obviously reduces the production of NO without affecting the cell viability in LPS stimulated RAW macrophages, which was connected to the reduced expression of iNOS protein. In addition, RUT significantly inhibited the expression of COX-2, TNF-α and IL-1β in activated macrophages. A previous study is corroborated with our results that an ethanolic extract of E. rutaecarpa inhibits NO production and iNOS expression in LPS-activated microglial and BV2 cells. Another study has also found a similar finding where it shows xanthotoxin, a furanocoumarin compound, potently inhibits the production of NO and the expression of iNOS, COX-2, TNF-α, and IL-1β, without affecting the viability in LPS stimulated RAW macrophages.

The PI3K/Akt signaling pathway is primarily accredited to control the activation of inflammatory cell response and the release of inflammatory mediators to play an imperative role during the chronic inflammatory response [28]. PI3K/Akt signaling facilitates various cellular events, such as proliferation, apoptosis, differentiation, and migration. Similarly, the MAPK signaling pathway is accountable for conveying signals toward the nucleus in response to several activators. This pathway controls a wider range of cellular events such as growth and stress. Studies have found that suppression of MAPK pathway is effective in inhibiting in vivo inflammation in mice and rabbit models [29,30]. Former studies have described that natural products, such as diarylheptanoid [31], capsaicin [32], and sesquiterpene lactone [33], inhibit iNOS and COX-2 by controlling MAPK signaling pathways. Moreover, luteolin, a yellow dye lavone compound, was noticed to regulate MAPK pathway and, thus, reserved the IL-1β-induced JNK and p38MAPK activation in SW982 cells [34]. This study shows that RUT has been effectively inhibited JNK and p38 MAPK phosphorylation in a concentration dependent manner in comparison with the effect on ERK phosphorylation, as RUT is only effective on its higher concentration.

NF-κB, a major transcription factor, plays vital roles in innate immunity and inflammation [35]. This transcription factor has been extensively approved as a crucial player in numerous processes associated with the stimulation of inflammation [36]. The NF-κB has also been found to facilitate several other signaling pathways and molecules in regulating inflammatory mediators during the process of inflammation. NF-κB activation increases the levels of pro-inflammatory cytokines; thus, inhibiting NF-κB pathway could be an ideal target for the anti-inflammatory drug developments. A recent study was found in the LPS stimulated Rat-1 fibroblast that luteolin inhibited the linkage between the NF-κB p65 and transcriptional coactivator and suppressed the NF-κB transcriptional activity [37]. Another study has also found a flavonoid compound quercetin inhibits LPS activated inflammatory responses in mononuclear cells by inhibiting the NF-κB signaling pathways [38].

Previous studies have shown that curcumin hinders the DNA binding action of NF-κB via suppressing of IκBα phosphorylation [39,40]. Jin et al. established that curcumin displays anti-inflammatory activities in LPS activated BV2 microglia cells by inhibiting the NF-κB signaling pathway, especially interfering to the nuclear translocation of p65 [41]. As our result remarkably found that RUT potently inhibited NF-κB signaling pathways, such as inhibiting the phosphorylation of IκBα and NF-κB p65 phosphorylation. It also effectively inhibits NF-κB p65 nuclear translocation as evidenced by the results of western blotting in the nuclear extract and confocal image analysis in RAW cells. Collectively, these results clearly indicate that RUT could be a valuable natural product for the management of inflammatory situations via inhibiting pro-inflammatory mediators by regulating PI3K/Akt and MAPK-mediated inhibition of NF-κB signaling pathways.

Src/FAK signaling pathway can be activated by the internalization of integrin β1, and participates in cell migration. The expression or activation of Src could replicate the migratory ability of macrophage [23]. A study by Miao et al., in 2016, noticed that FAK and Src inhibitors could efficiently reduce the migratory ability of macrophages induced by hydrogen sulfide (H_2_S) [42]. Quercetin, a natural flavonoid has demonstrated to inhibit LPS-induced macrophage migration by inhibiting NO production and suppressing the FAK pathway [43]. In the present study, LPS treatment significantly affected the migration of RAW macrophages by increasing their proliferation, whereas RUT potently reduces its migration as shown in the Figure 7A. RUT treatment alone almost had similar activity to that of control cells. This result suggests that LPS activates macrophage migration via the Src FAK/pathways and RUT blocks this pathway in accordance with reducing the migration ability. Maa et al., in 2010, demonstrated the consistent result with this study that activation of both Src and FAK enhances macrophage migration and butyrate, a short-chain fatty acid suppressed these activations followed by macrophage migration in LPS induced RAW264.7 and rat peritoneal macrophages [44]. This result may offer a fundamental model to explain how RUT-mediated inhibition of LPS-triggered macrophage migration. Though further study of the principal mechanisms is required, we concluded this study that RUT may offer a protective mechanism by controlling multiple signaling cascades; hence, this alkaloid compound could be used as a drug candidate for treating inflammation-mediated diseases.

## 4. Materials and Methods

### 4.1. Chemicals and Reagents

Fetal bovine serum (FBS), Dulbecco’s modified Eagle medium (DMEM), l-glutamine penicillin/streptomycin, and anti-α-tubulin monoclonal antibodies (mAbs) were purchased from Invitrogen (Thermo Fisher Scientific, Waltham, MA, USA). LPS (Escherichia coli 0127:B8), 3-(4,5-dimethylthiazol-2-yl)-2,5-diphenyltetrazolium bromide (MTT), and dimethyl sulfoxide (DMSO) were purchased from Sigma-Aldrich (St. Louis, MO, USA). Anti-Lamin B1 and anti-iNOS polycloncal antibody (pAb) were purchased from Santa Cruz Biotechnology (Dallas, TX, USA). The anti-TNF-α, anti-phospho-c-JNK (Thr^183^/Tyr^185^), anti-phospho-p44/p42 ERK (Thr^202^/Tyr^204^), anti-phospho-p38 MAPK (Thr^180^/Tyr^182^) pAbs, anti-phospho-p65 (Ser^536^), anti-phospho-Src family (Tyr^416^), and anti-phospho-focal adhesion kinase (FAK) (Tyr^397^) mAbs were purchased from Cell Signaling (Danvers, MA, USA). Anti-IL-1β pAb was purchased from BioVision (Milpitas, CA, USA). Horseradish peroxidase (HRP)-conjugated donkey anti-rabbit immunoglobulin G (IgG), and sheep anti-mouse IgG were purchased from Amersham (Buckinghamshire, UK). The Western blotting detection reagent of enhanced chemiluminescence (ECL) and Hybond™-P polyvinylidene difluoride (PVDF) blotting membranes were purchased from GE Healthcare Life Sciences (Waukesha, WI, USA).

### 4.2. RAW 264.7 Cell Cultivation

RAW 264.7 cells were purchased from ATCC (ATCC number: TIB-71). The cells were cultured in DMEM supplemented with 10% FBS and 100 U/mL penicillin G and 100 mg/mL streptomycin at 37 °C in a humidified atmosphere of 5% CO_2_/95% air.

### 4.3. Cell Viability Assay

RAW 264.7 cells (2 × 10^5^ cells per well) were seeded into 24-well culture plates with DMEM containing 10% FBS for 24 h. The cells were treated with various concentrations of rutaecarpine (10–100 μM) or solvent control (0.1% DMSO) for 20 min, and then stimulated with LPS (1 μg/mL), or left unstimulated, for 24 h. Cell viability was measured by using MTT assay. The cell viability index was calculated as follows: (absorbance of treated-cells/absorbance of control cells) × 100%. The absorbance of samples was determined at 570 nm by an MRX absorbance reader (Dynex Technologies, Chantilly, VA, USA).

### 4.4. Determination of Nitric Oxide Production

To determine NO production, the level of nitrite/nitrate, stable oxidative end products of nitric oxide, was measured as previously described with minor modifications. Then, 8 × 10^5^ RAW 264.7 cells were seeded into 6-cm dishes with DMEM containing 10% FBS for 24 h. The cells were treated with rutaecarpine (10 and 20 μM) or solvent control (0.1% DMSO) for 20 min and then stimulated with LPS (1 μg/mL), or left unstimulated, for 24 h. These conditioned supernatants were collected and mixed with equal volumes of Griess reagent (1% sulphanilamide and 0.1% naphthalenediamine dissolved in 2.5% phosphoric acid). The absorbance of samples was determined at 540 nm by an MRX absorbance reader. The concentrations of nitrite/nitrate were calculated by a standard curve performed through the linear regression of absorbance measurements of standard solutions (sodium nitrite dissolved in the same culture medium).

### 4.5. Separation of Cytoplasmic and Nuclear Extracts

RAW 264.7 cells (8 × 10^5^ cells per dish) were treated with 0.1% DMSO or 20 μM rutaecarpine with or without LPS stimulation for 30 min in 6 cm dishes and were maintained in a humidified atmosphere. Subsequently, the cells were harvested, and cytoplasmic and nuclear proteins were extracted using the NE-PER kit (Thermo Fisher Scientific, Waltham, MA, USA) according to the manufacturer’s instructions. Lamin B1 and α-tubulin were used as internal controls for the nucleus and cytosol, respectively.

### 4.6. Immunofluorescence Staining Assay

RAW 264.7 cells (5 × 10^4^ cells per well) were cultured on cover slips in 6-well plates and treated with 0.1% DMSO or 20 μM rutaecarpine with or without LPS stimulation for 30 min. The cells were washed with phosphate-buffered saline (PBS) and fixed with 4% paraformaldehyde in PBS for 10 min at room temperature. After incubation, the cells were permeabilized with 0.1% Triton X-100 for 10 min and blocked with 5% BSA for 30 min. The cells were incubated with primary antibodies overnight at 4 °C, subsequently washed 3 times with PBS, and incubated with secondary antibodies for 1 h at room temperature. The samples were stained with 4,6-diamidino-2-phenylindole (DAPI, 30 μM) and mounted using a mounting buffer (Vector Laboratories) on a glass slice. The samples were detected under a Leica TCS SP5 confocal spectral microscope imaging system using an argonor krypton laser (Mannheim, Germany).

### 4.7. Western Blotting

A Western blotting analysis was performed to determine the protein expression in cells and tissue homogenates, as previously described. RAW 264.7 cells (8 × 10^5^ cells/dish) were seeded on 6-cm dishes with DMEM containing 10% FBS for 24 h. The cells were pretreated with rutaecarpine or 0.1% DMSO for 20 min and either stimulated with LPS (1 μg/mL) or left unstimulated, according to the experimental design. Subsequently, the proteins from the cells and liver tissues were extracted using lysis buffer. The extracted protein samples (50 μg) were applied to sodium dodecyl sulphate (SDS)-polyacrylamide gel electrophoresis, and the separated proteins were then electrophoretically transferred onto PVDF membranes (0.45 μm). The membranes were blocked with 5% skimmed milk in TBST buffer (10 mM Tris-base, 100 mM NaCl, and 0.01% Tween 20) for 30 min. The membranes were incubated with the targeting primary antibodies against iNOS, TNF-α, IL-1β, phospho-p38 MAPK, phospho-c-JNK, phospho-p44/p42 ERK, IκBα, phospho-p65, phosphor-Src, and phosphor-FAK for 2 h, and then subjected to HRP-conjugated donkey anti-rabbit IgG or sheep anti-mouse IgG for 1 h at room temperature. The ECL system was used to detect the immune-reactive bands. The densitometry of protein bands was performed by the Biolight Windows Application, V2000.01 (Bio-Profil, Vilber Lourmat, France).

### 4.8. Wound Healing Migration Assay

Raw 264.7 cells were grown until they had reached about 80% confluence. Then a wound was created in the cell monolayer by a scratching a vertical line at the center of each well using a sterile 200 μL pipette tip. Cells were washed twice with PBS to remove debris, followed by incubation with 1 μg/mL LPS in the absence or presence of rutaecarpine in DMEM containing 10% FBS for 24 h. The wound area was photographed at time 0 and designated incubation times under a light microscope (Nikon, Tokyo, Japan). Cells migrated off the scratch edges toward the wound area were counted using ImageJ 1.47v software. To quantify cell migration, images of the initial wounded monolayers were equated to the corresponding pictures of cells at later time points.

### 4.9. Statistical Analysis

The results are presented as the means ± standard error (SEM) and are accompanied by the number of observations (*n*). Data were assessed using one-way analysis of variance (one-way ANOVA). If one-way ANOVA revealed significant differences among the group means, a subsequent comparison of Newman-Keuls method was performed. A *p* value < 0.05 was considered as a statistically significant difference. The image of all the western blotting data for the expression of targets and internal proteins have been normalized with the control group. The relative density values from the treated groups were determined by dividing the optical density values from these treated groups by the value of the control group after each value was normalized to α-tubulin. The experiments were repeated at least 4 times/treatment, but the blot density from the control group per experiment was always set as 1.

## 5. Conclusions

According to the network pharmacology, the underlying mechanism of rutaecarpine in anti-inflammatory treatment involves the regulation of signaling pathways and targets in several biological progressions. The anti-inflammatory effects of RUT in macrophage cells appear to be facilitated by the inhibition of the release of proinflammatory mediators and regulation of NF-κB, MAPK, and PI3K-Akt signaling pathways. Stimulatingly, this is the first study investigated the Src/FAK-meditated cell migration prevention activity of RUT in LPS induced RAW 264.7 macrophages. Altogether, the gained results of this study may recommend that RUT can be widely used as an exceptional natural substance for the treatment of inflammatory diseases.

## Data Availability

All data generated or analyzed during this study are included in this published article.
